# Salvage Antiperistaltic Left Colon Conduit for Caustic Esophageal Stricture: A Viable Alternative Route in a Hostile Mediastinum

**DOI:** 10.7759/cureus.102155

**Published:** 2026-01-23

**Authors:** Neha I Mulgund, Aravind Ramkumar, Rajath Shetty, Trilok N Y, Jawad Uddin

**Affiliations:** 1 General Surgery, ESIC Medical College and PGIMSR and MH, Bengaluru, IND; 2 General Surgery, Malavya Hospital, Bengaluru, IND

**Keywords:** antiperistaltic colon conduit, caustic esophageal stricture, colonic conduit, conduit necrosis, corrosive injury, esophageal reconstruction, esophageal stricture

## Abstract

Corrosive ingestion is a serious condition that can result in multiple esophageal strictures, often necessitating complex surgical reconstruction when endoscopic management fails. Colonic interposition is an established option for esophageal replacement, but conduit necrosis remains a potentially fatal complication. We present details of a rare salvage antiperistaltic left colon conduit for a patient with corrosive esophageal stricture, who failed endoscopic dilatation and a right ileocolonic conduit.

## Introduction

Corrosive ingestion leads to severe chemical injury of the upper gastrointestinal tract, most commonly affecting the esophagus and stomach. The extent of tissue destruction depends on the type, concentration, and duration of contact with the corrosive agent. Alkaline agents typically cause liquefactive necrosis and deeper transmural damage, while acids generally result in coagulative necrosis with eschar formation, which may limit further penetration. The subsequent healing process often leads to fibrosis, scarring, and the formation of long-segment esophageal strictures. The management of corrosive-induced strictures depends on their location, extent, and response to endoscopic dilatation. Serial endoscopic balloon dilatation remains the cornerstone of initial therapy. However, in cases where the stricture is multiple, complex, or unresponsive to dilatation, surgical reconstruction becomes necessary to restore alimentary continuity and adequate nutrition. While the stomach remains the preferred conduit, surgeons may draw from an armamentarium of alternative approaches when conventional reconstruction proves unfeasible [1]. Several conduits have been described for esophageal substitution, including the stomach, colon, and jejunum. Among these, the colon is often the conduit of choice due to its appropriate length, stable lumen, resistance to acid reflux, and well-preserved vascular network, which allows for flexible mobilization. Either the right (ascending) or left (descending) colon may be used, depending on vascular anatomy, reach, and the surgeon’s preference. However, colonic conduit necrosis, though relatively uncommon (incidence 2%-10%), remains one of the most catastrophic postoperative complications. It may result from inadequate vascularization, conduit tension, or technical errors in conduit orientation and is associated with significant morbidity and mortality due to sepsis, mediastinitis, or anastomotic leakage. Early recognition and prompt surgical intervention are critical for patient survival. We report a rare and challenging case of corrosive-induced esophageal stricture in which an initial ascending colonic conduit failed due to venous congestion. In this rare and challenging situation, we proceeded with salvage reconstruction using an antiperistaltic left colonic conduit based on the middle colic artery, as this was the only viable option available to us. To the best of our knowledge, this is the first case of salvage operation using this conduit, though there is a publication reporting the technique of procedure in a primary setting [2]. This case highlights the importance of intraoperative vascular assessment, vigilance for early conduit ischemia, and adaptability in surgical planning to achieve favorable outcomes in complex esophageal reconstructions.

## Case presentation

A 46-year-old male with a history of accidental corrosive ingestion was initially evaluated elsewhere, where upper gastrointestinal (GI) endoscopy revealed a Zargar grade IIIb corrosive injury with eschar formation involving the fundus and body of the stomach. Ultimately, endoscopy is considered the cornerstone in the diagnosis, prognostication, and guidance for the management of caustic ingestions. Various endoscopic grading systems are available, and Zargar’s classification is one of the most commonly used [3]. He was subsequently referred to our center for further management. Imaging studies (CT neck, thorax, and abdomen) demonstrated no evidence of esophageal or gastric perforation and no signs of mediastinitis. A feeding jejunostomy was performed to facilitate enteral nutrition. The patient was also managed with parenteral antibiotics and oral antacids, and he responded well to conservative management during the acute phase. However, over the ensuing five months, he developed esophageal strictures, a recognized late complication of severe corrosive injury.

**Figure 1:**
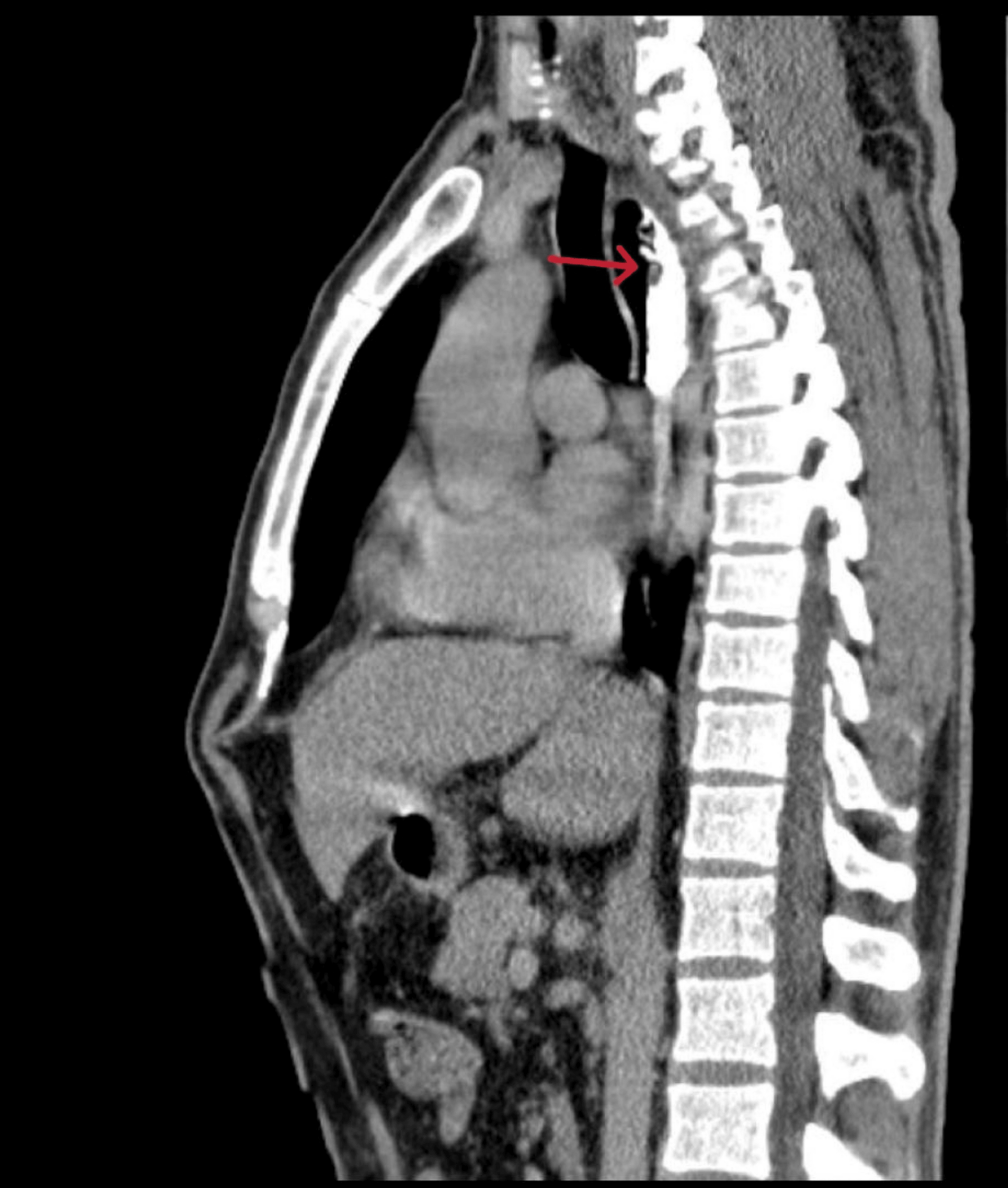
CT thorax showing contrast-filled upper esophagus with mucosal irregularity

**Figure 2:**
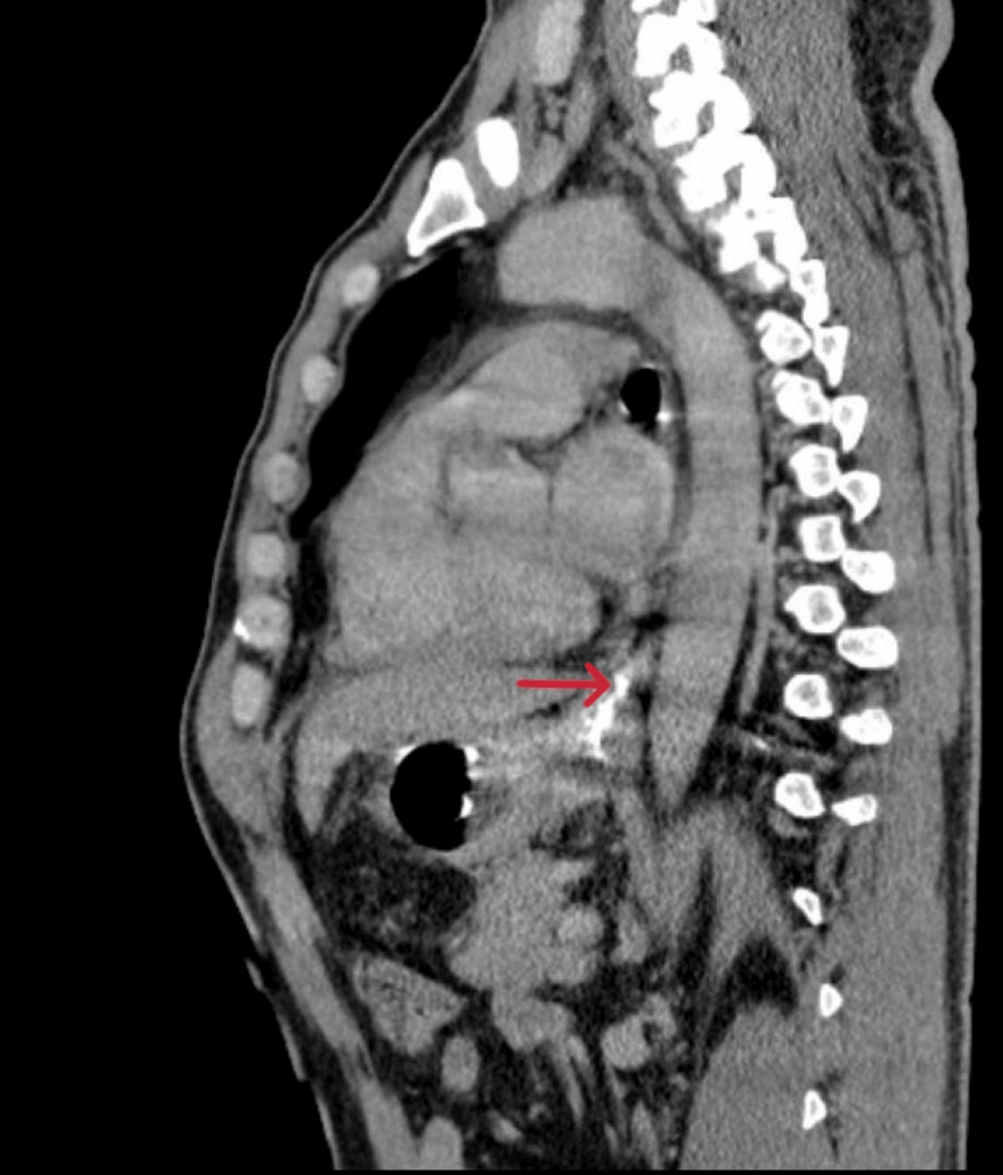
Contrast CT showing lower esophageal stricture

**Figure 3:**
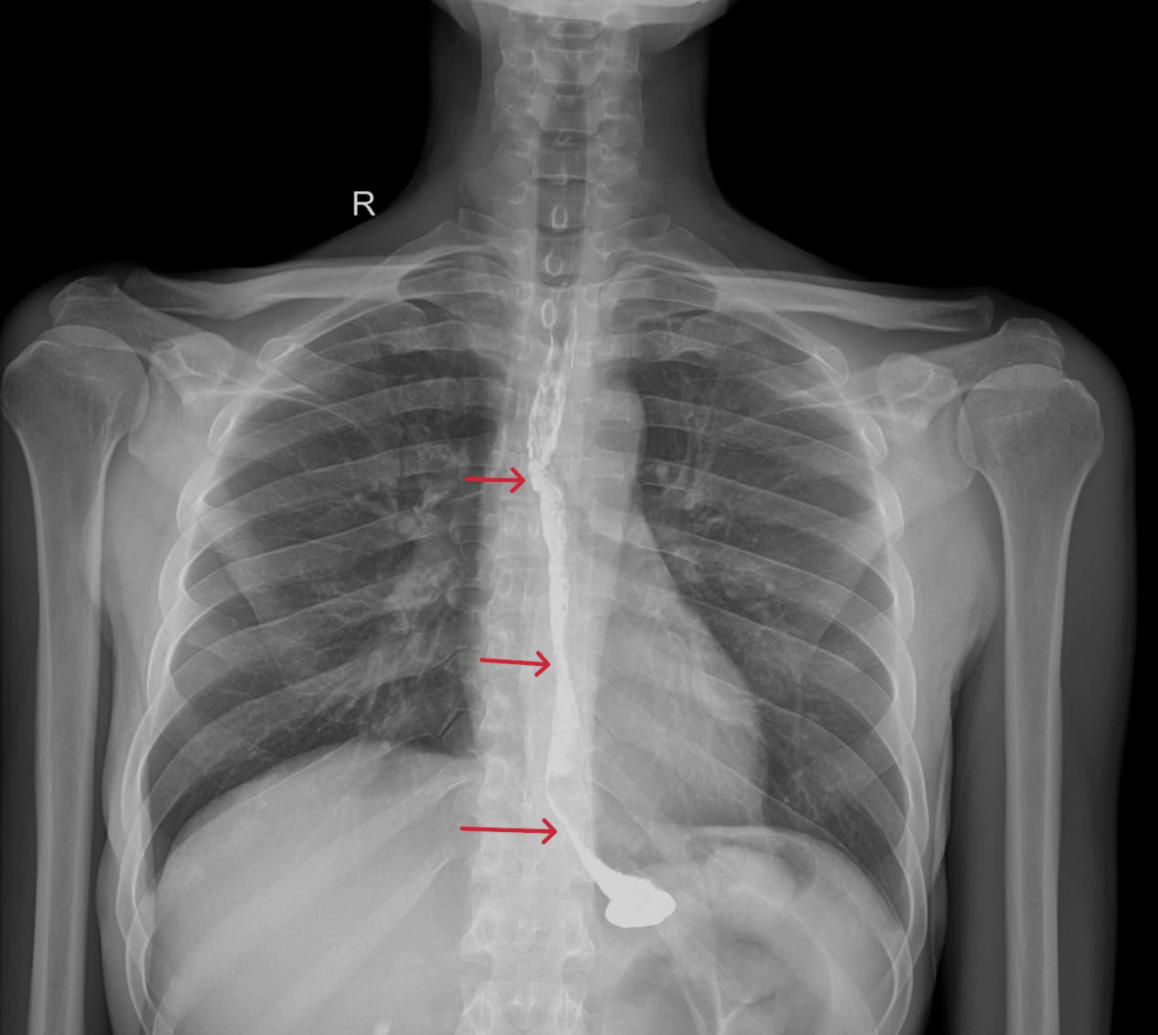
Barium swallow showing multiple esophageal strictures

**Figure 4:**
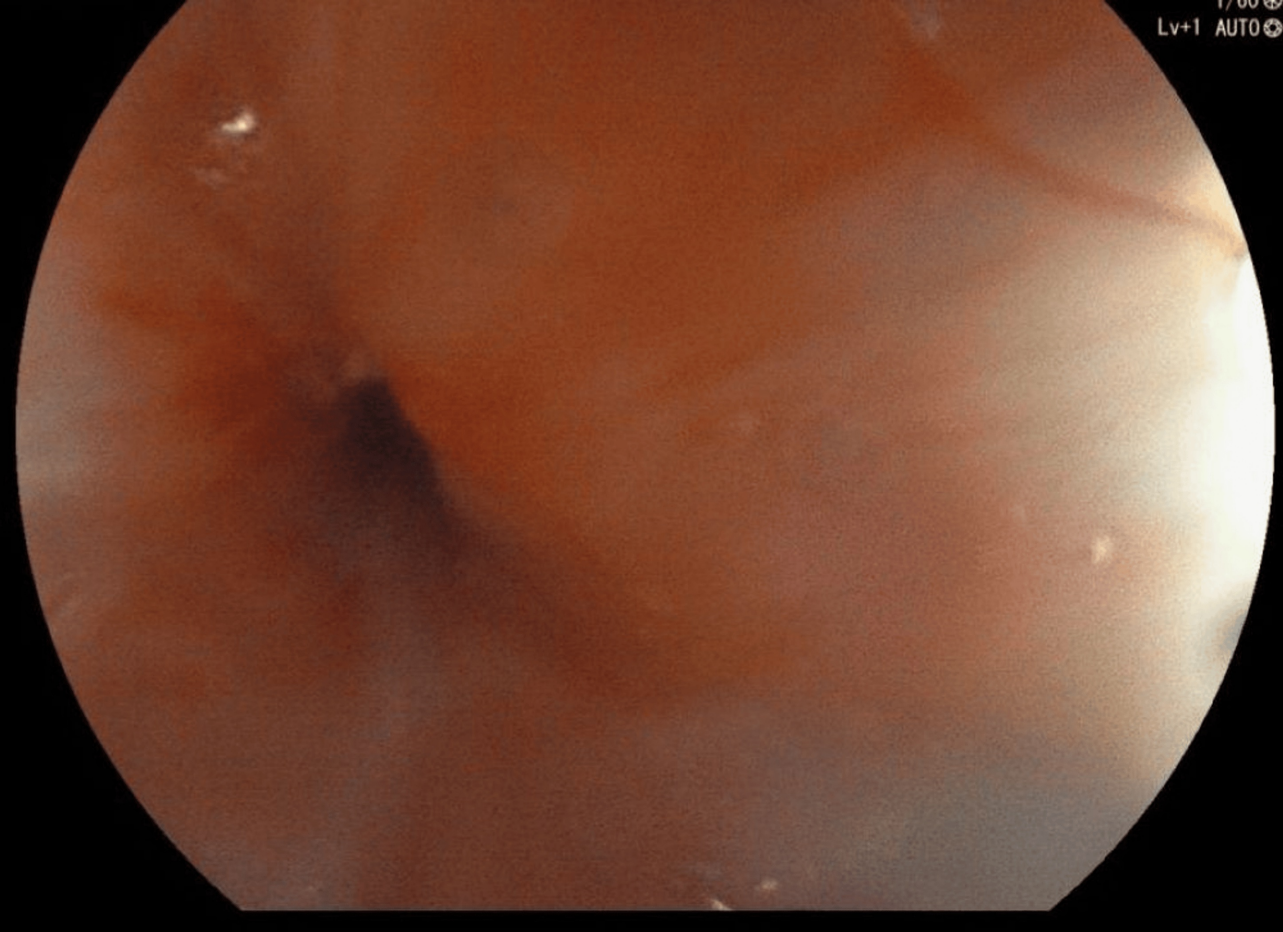
Upper GI endoscopy showing tight esophageal stricture GI, gastrointestinal.

He underwent contrast-enhanced CT (Figures 1, 2) and barium swallow (Figure 3) and upper GI endoscopy (Figure 4), which revealed a stricture just beyond the post-cricoid region and mid-thoracic esophagus. The endoscope could not be passed beyond the post-cricoid stricture.

**Figure 5:**
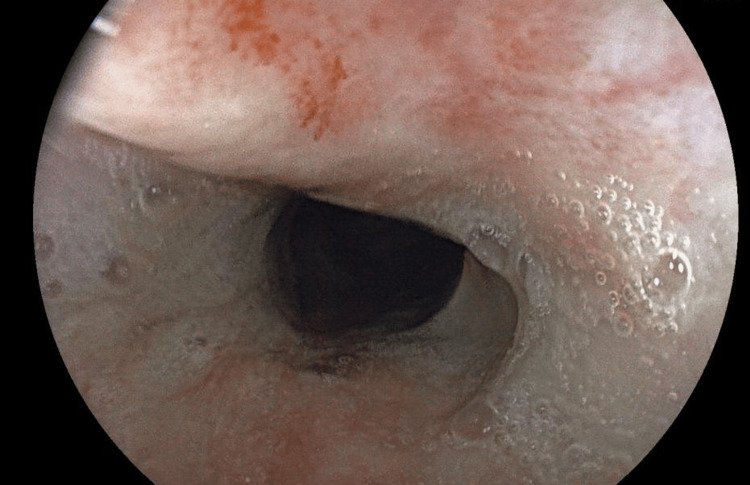
Upper GI endoscopy after dilatation of post-cricoid stricture GI, gastrointestinal.

The patient underwent three sessions of dilatations with Savary-Gilliard bougies and pneumatic balloon dilatation for further post-cricoid stricture. On one such attempt, the endoscope could be passed beyond only to reveal a stricture at the mid-thoracic esophagus. In spite of multiple dilatations, the patient developed recurrent stricture in the post-cricoid region (Figure 5). Given the failure of multiple dilatation attempts and the presence of double-level strictures (post-cricoid and distal esophagus), the case was deemed unsuitable for further endoscopic management. After multidisciplinary evaluation involving the surgical and gastroenterology teams, a decision was made to proceed with definitive surgical reconstruction.

The surgical plan was to do staged reconstruction with initial stricturoplasty with pedicled pectoralis major or radial forearm free flap for the post-cricoid stricture, followed by colonic conduit for the midthoracic esophageal stricture. 

Under general anesthesia, the patient was positioned in a supine position, and a right-sided oblique neck incision, approximately 6 cm in length, was made medial to the sternocleidomastoid muscle to expose the cervical esophagus. Careful neck dissection was performed to identify and isolate the cervical esophagus. On-table endoscopy confirmed a tight post-cricoid esophageal stricture, which was incised longitudinally until the lumen was reached. A 14 French Ryle’s tube was passed retrogradely through the opened segment to confirm proximal patency of a small segment of uninvolved esophagus just below the post-cricoid region. An attempt was then made to advance Ryle’s tube distally through the distal esophageal lumen, but it was not possible to confirm the presence of a second tight segment continuing from the neck into the mediastinum. Given the impassable distal stricture, a decision was made to perform a transthoracic esophagectomy and gastric or colonic pull-up with anastomosis with the remnant esophagus. The thoracic esophagus was mobilized through the posterolateral thoracotomy incision from the subclavian vessels to the esophageal hiatus. The chest was closed with the intercostal drain, and the abdomen was explored via midline laparotomy, revealing scarred stomach and a viable colon suitable for interposition. The stomach was transected at the fundus, the cervical esophagus was divided, the Ryle's tube was sutured to the distal cut end of the esophagus, and the entire esophagus was delivered into the abdomen with the tube traversing from the neck to the abdomen through the posterior mediastinum.

**Figure 6:**
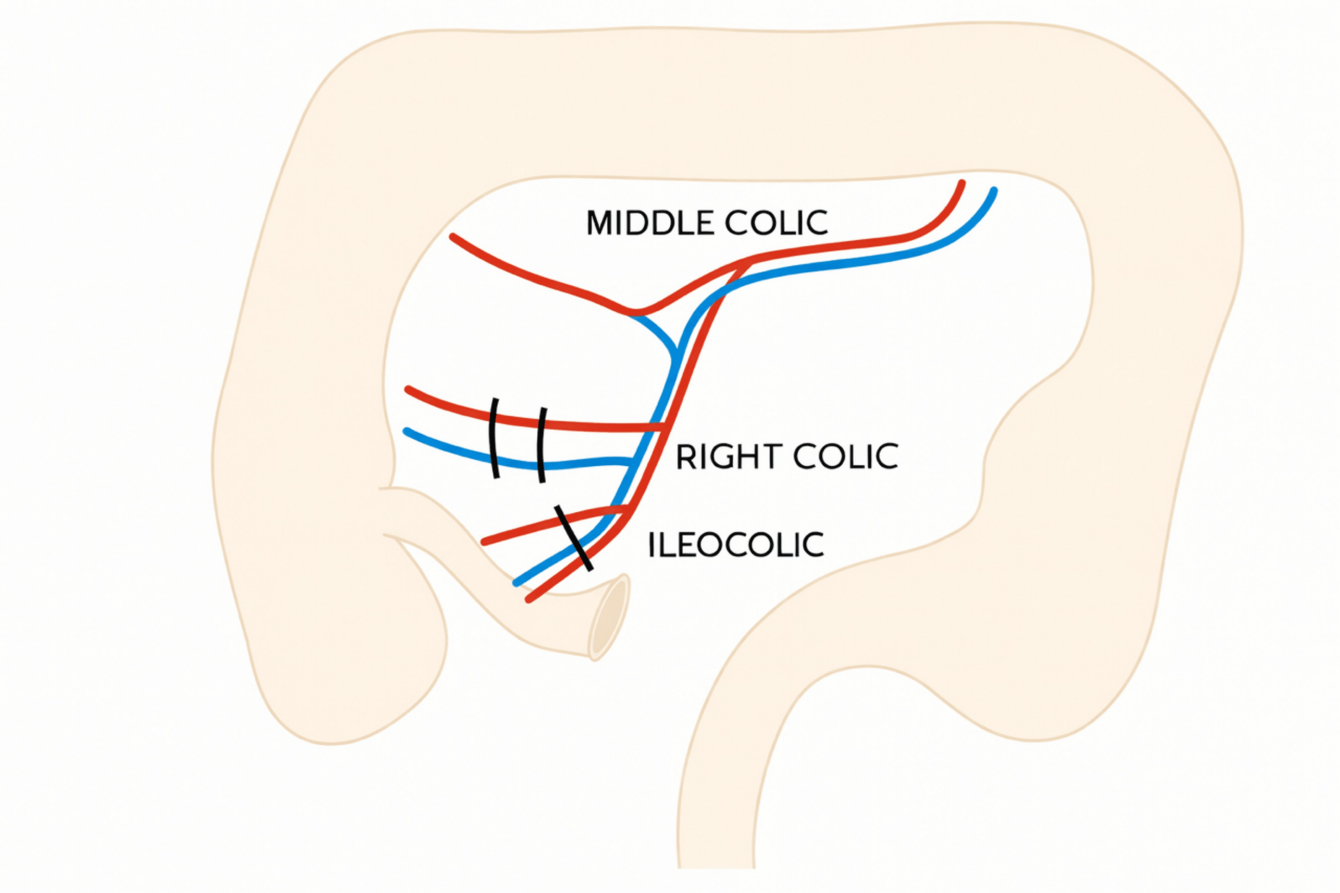
Line diagram showing the ligated ileocolic and right colic vessels with transection of the transverse colon to harvest the right colonic conduit Image credits: Dr. Neha Mulgund

An isoperistaltic ascending colonic conduit was then constructed based on the middle colic artery after ligating the ileocolic and the right colic artery and transposed through the posterior mediastinal route for esophageal replacement (Figure 6).

**Figure 7:**
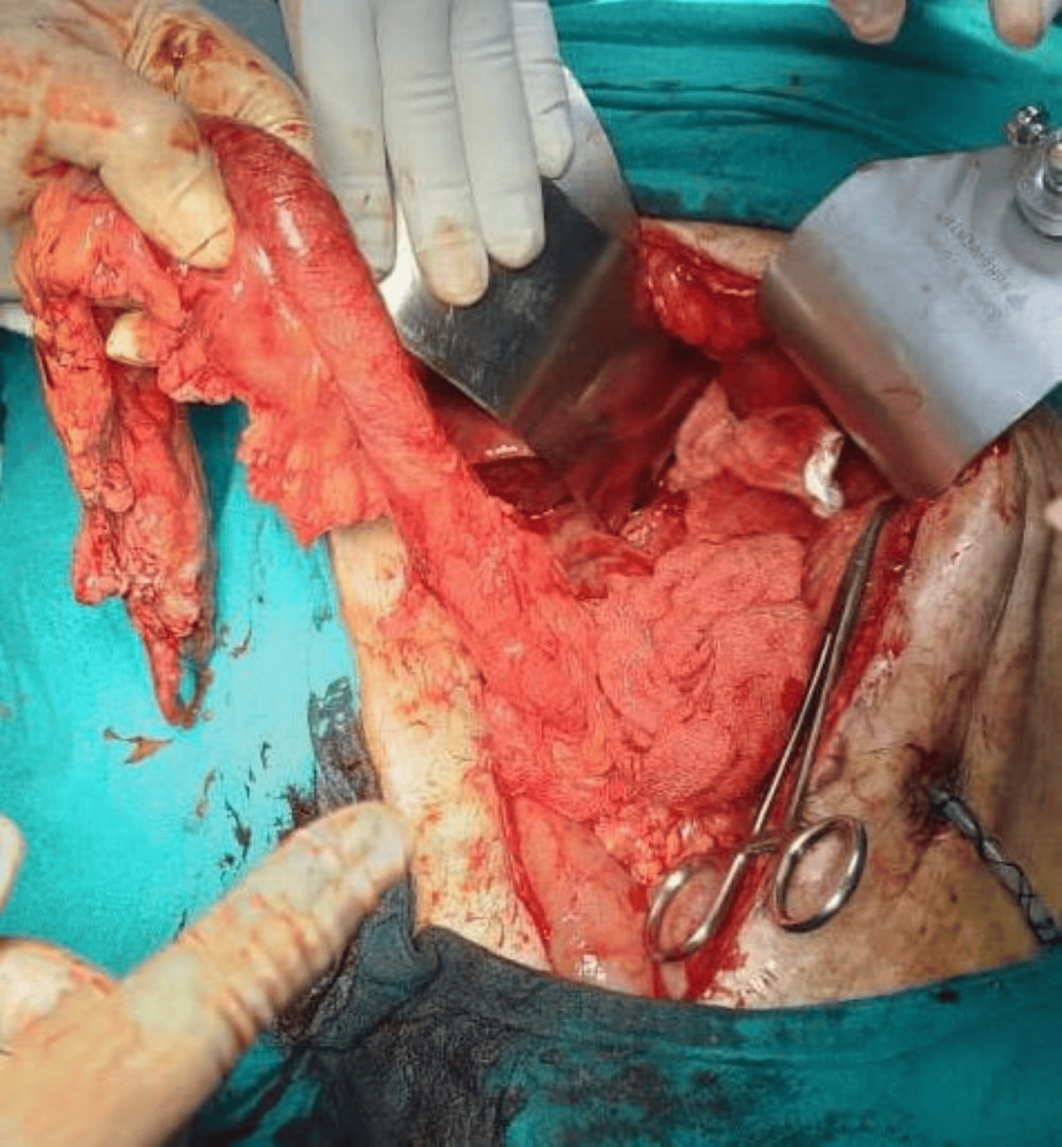
Left colonic conduit after ligating left colic artery and branches of sigmoidal arteries and left colon mobilization

The reconstruction included colojejunal and ileocolic anastomoses. Appendectomy was also carried out. At the end of the surgery, congestion of the proximal end of the ileocolonic conduit was noted (ileum and ascending colon). However, on scoring the serosa, there was bright bleeding. Hence, the decision was made to proceed with the colonic conduit with esophagocolic anastomosis. The conduit was oriented in an isoperistaltic fashion and transposed through the posterior mediastinal route within a sterile protective sleeve to the cervical field. The patient tolerated the procedure well. However, on postoperative day two, a hematoma was noted at the neck incision, and sutures were removed, which revealed conduit ischemia. Immediate re-exploration was undertaken. Intraoperative findings confirmed necrosis of the ileum and ascending colon part of the conduit. The conduit was pulled back into the abdomen through the laparotomy, the necrosed part was excised, and the field was thoroughly irrigated. Subsequently, the options available were to reconstruct using the left colon either isoperistalitic, based on the left colic artery, or antiperistalitic, based on the middle colic artery (Figure 7).

**Figure 8:**
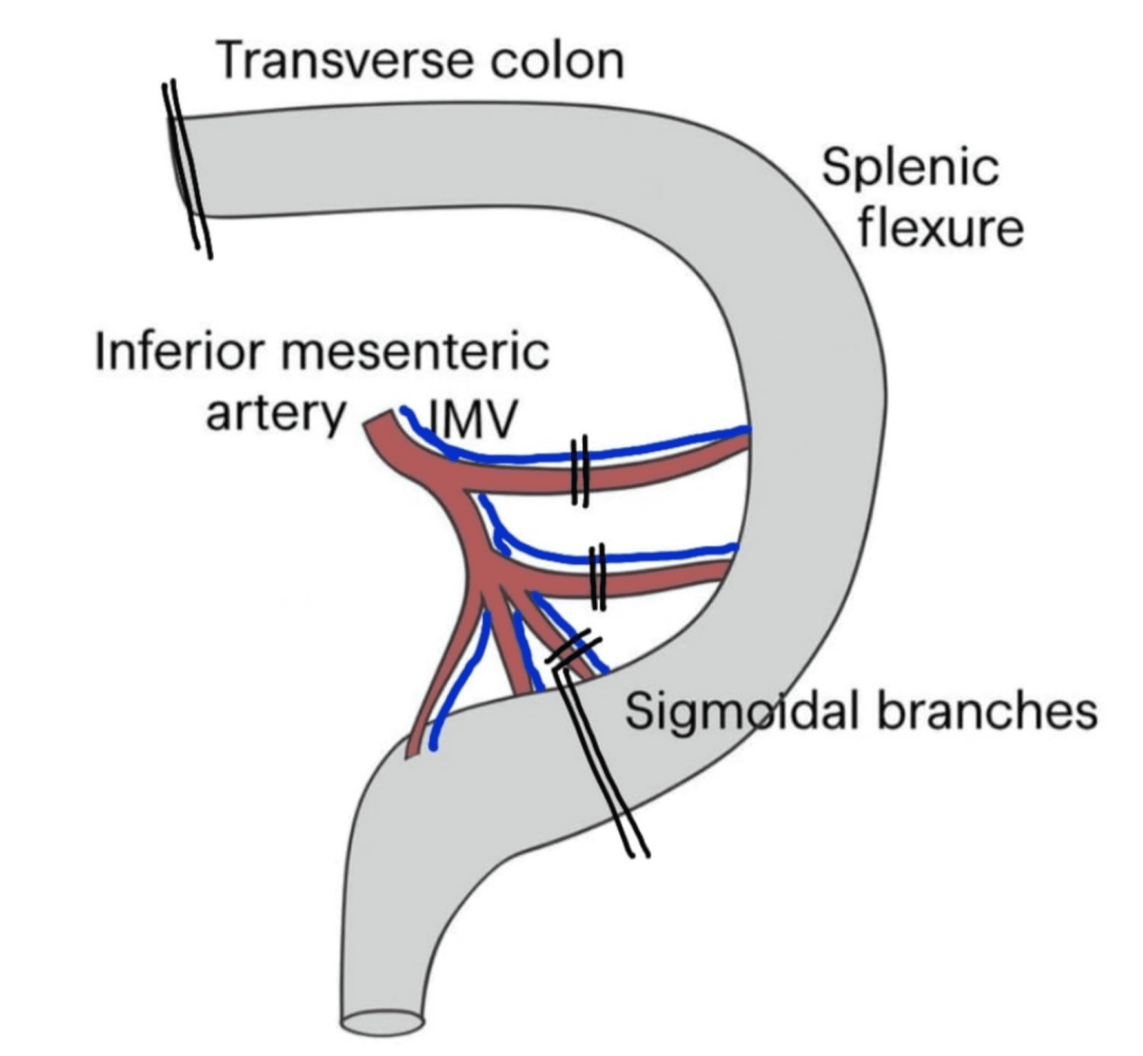
Left colonic conduit after ligating the left colic artery and branches of sigmoid artery IMV, inferior mesenteric vein. Image credits: Dr. Neha Mulgund

Middle colic artery branching was inspected and was found to have early branching, with the branches almost arising directly from the superior mesenteric vessels. Hence, a decision was made to use an antiperistaltic colonic conduit (Figure 8).

Bulldog clamps were placed on the left colic artery and the proximal two sigmoid arteries after complete mobilization of the left colon. Measurements were taken to check whether the conduit would reach the pharynx. The vascularity of the colon was good.

**Figure 9:**
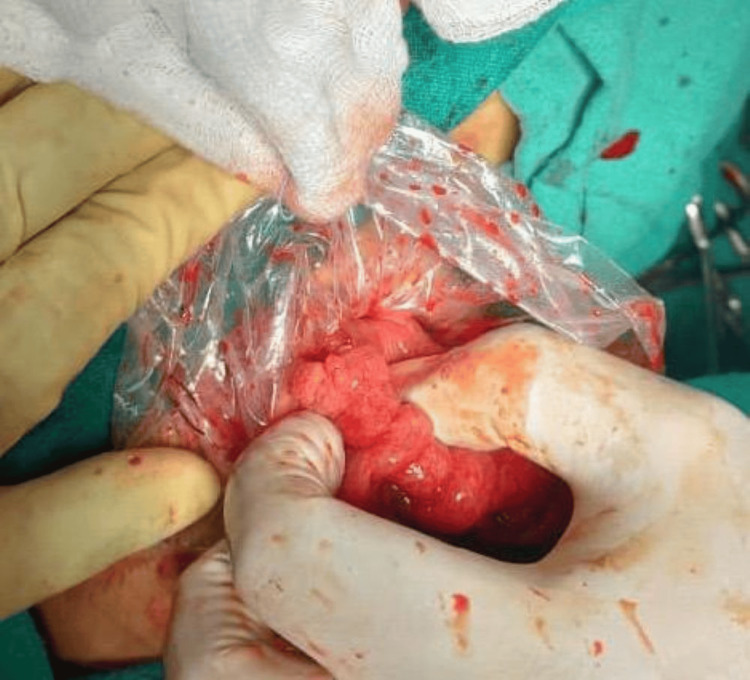
Conduit oriented in an antiperistaltic fashion and transposed through the posterior mediastinal route within a sterile protective sleeve to the cervical field

Reconstruction was performed using a left colonic conduit based on the middle colic artery, after ligation of the above-mentioned branches. The conduit was oriented in an antiperistaltic fashion and transposed through the posterior mediastinal route within a sterile protective sleeve to the cervical field (Figure 9).

**Figure 10:**
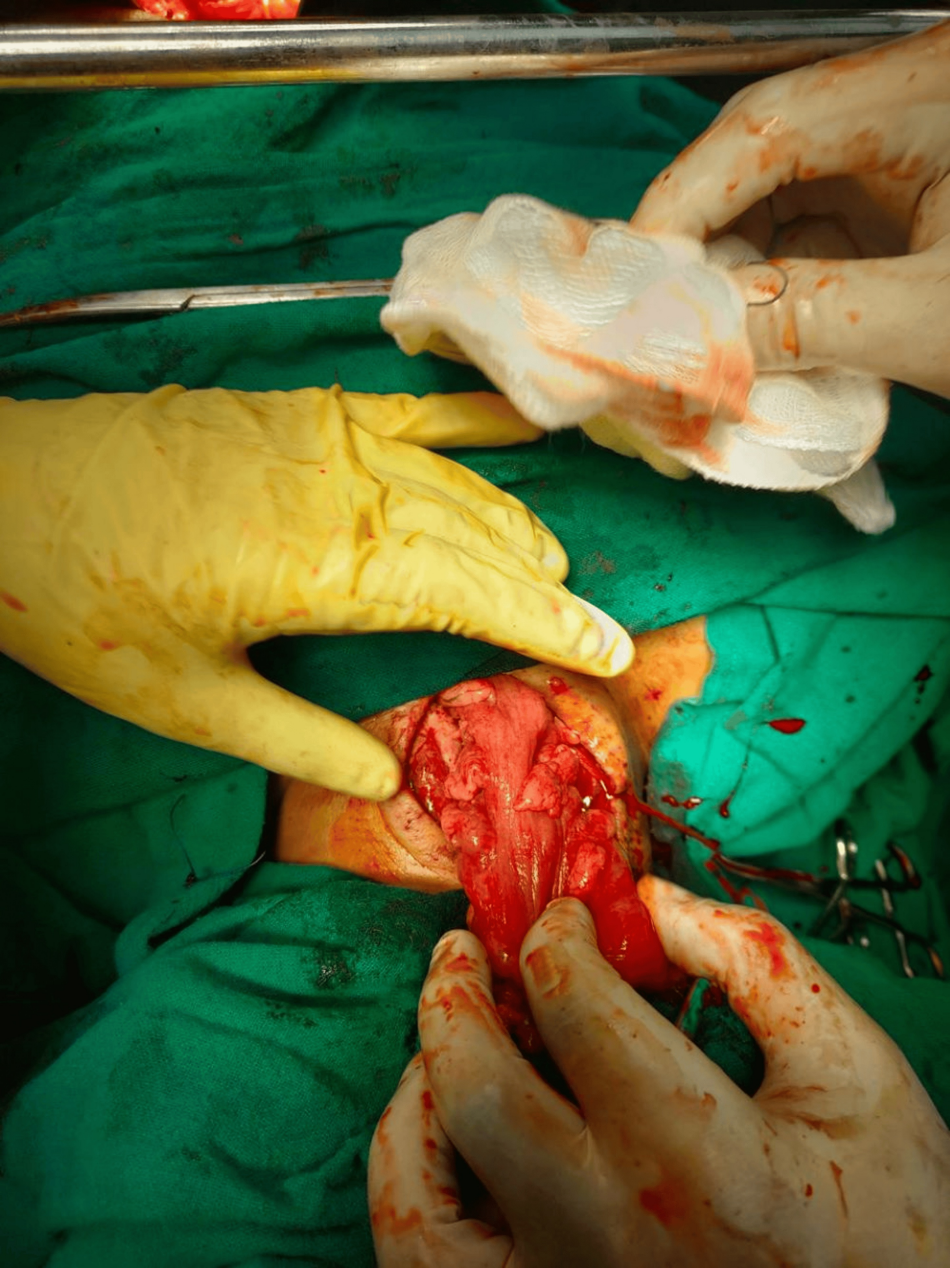
Tension-free cervical esophagocolic anastomosis

A tension-free cervical esophagocolic anastomosis was then completed (Figure 10).

**Figure 11:**
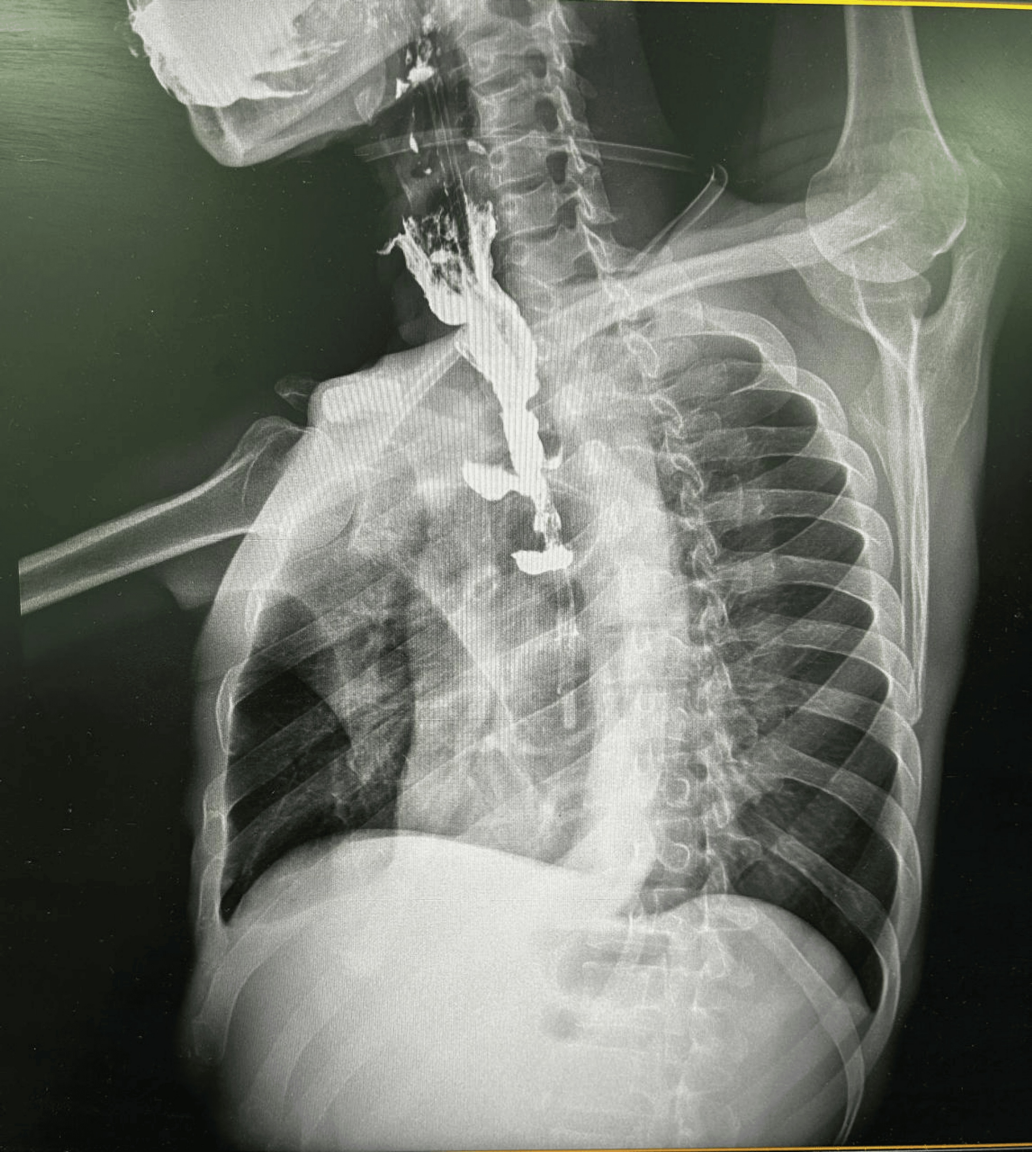
Post-operative barium swallow lateral view

**Figure 12:**
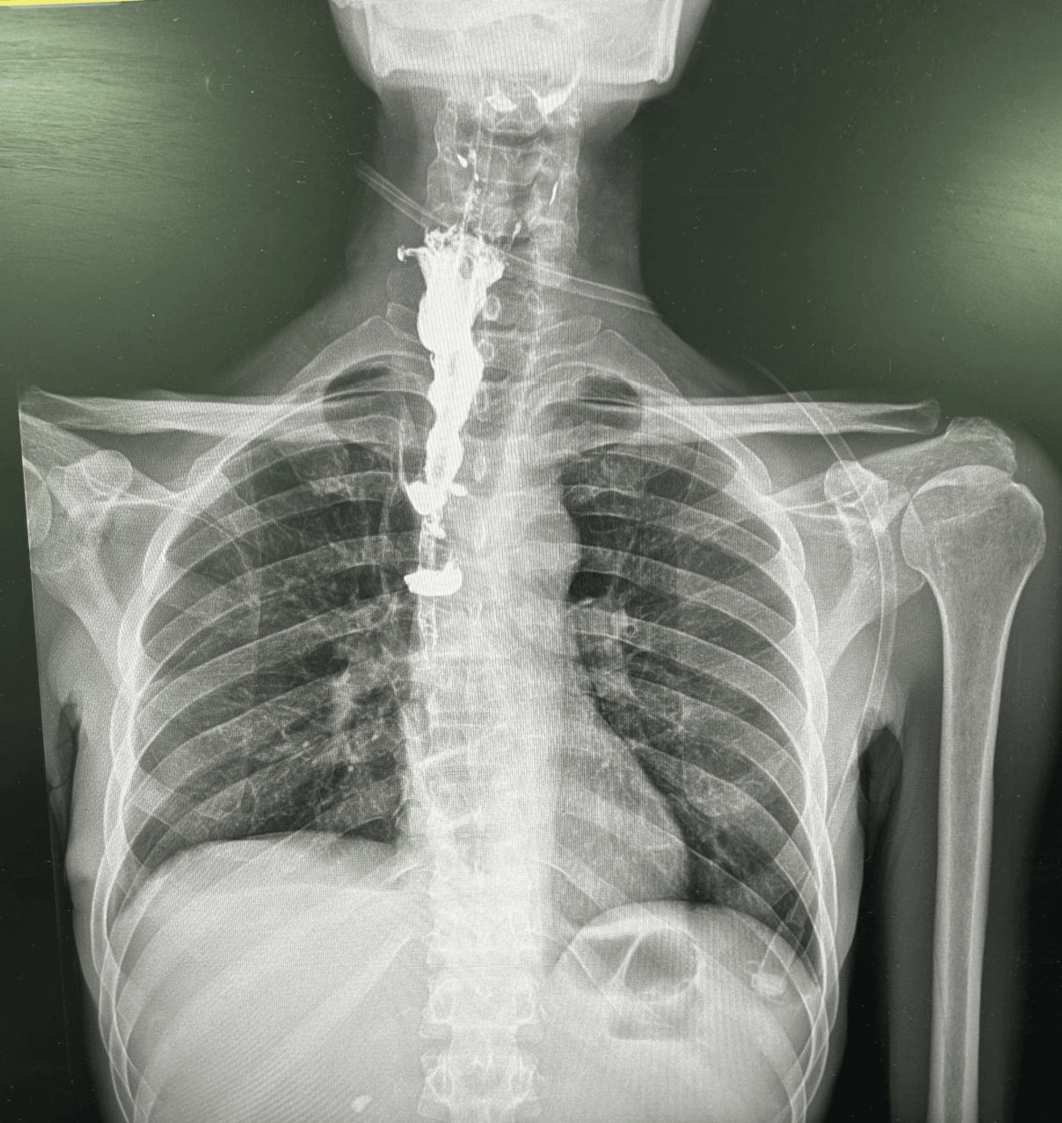
Post-operative barium swallow postero-anterior view

The remaining bowel continuity was restored with colojejunal and ileorectal anastomoses after dismantling the previous anastomoses. The newly constructed conduit appeared well vascularized with satisfactory pulsations and color. The patient tolerated the re-exploration well, was shifted to the surgical intensive care unit, had an uneventful postoperative recovery, and had a minor cervical anastomotic leak ( without systemic features of sepsis), which was managed conservatively and healed. The patient is on an oral regular diet, 4 weeks from the date of surgery, with complaints of reflux on and off (Figures 11, 12).

## Discussion

Corrosive injuries of the esophagus are among the most complex pathologies encountered in upper GI surgery. The severity of injury depends on the nature, concentration, and quantity of the corrosive substance ingested. Alkaline agents cause liquefactive necrosis that penetrates deeply, whereas acids induce coagulative necrosis that typically leads to more superficial but circumferential scarring [4]. The resulting fibrosis and stricture formation are often extensive, leading to dysphagia, aspiration, and nutritional compromise. Once established, corrosive strictures are challenging to manage due to the altered anatomy, dense adhesions, and limited tissue pliability. Endoscopic dilatation is the mainstay of treatment for short or single-segment strictures; however, in long- segment or multiple strictures, dilatation alone is frequently inadequate, necessitating definitive surgical reconstruction. The primary goal of surgical management is to restore alimentary continuity using a well-vascularized, tension-free, and durable conduit while minimizing morbidity and preserving swallowing function. Selecting the appropriate conduit and route of reconstruction plays a critical role in long-term success. Surgical options include partial or total esophagectomy with gastric pull-up or, preferably, colonic interposition. Gastric pull-up, in general, is quicker and requires only one anastomosis. However, the long-term functional outcome may decrease with the development of complications such as recurrence of stricture, bothersome reflux, and subsequent metaplasia over the anastomotic site. On the other hand, colon interposition is a more complex procedure requiring three anastomoses, albeit with a more stable long-term functional outcome. It is often associated with a lower incidence of stricture formation than gastric pull-up, hence its preferential use in the setting of a relatively spared and healthy stomach [5].

We followed the staged cervical management and mid-colon conduit principles described by Ananthakrishnan et al., who emphasized restoring a patent cervical esophageal lumen before definitive bypass in patients with complex post-cricoid or pharyngo-esophageal strictures. In their technique, stricturoplasty with a pectoralis major myocutaneous [6] or sternocleidomastoid flap [7] is used to reconstitute the upper esophagus before subsequent colonic replacement. This staged approach has been shown to provide a safe and reliable method for reconstruction in cases of severe corrosive or post-radiation strictures.

However, in our patient, the inability to find an adequate lumen in the distal esophagus after neck exploration prompted us to proceed with esophagectomy with a gastric/colon conduit. The presence of a scarred stomach on laparotomy drove us to harvest an ileocolonic pull-up. Colon interposition for esophageal substitution, usually performed when the stomach is not available, provides satisfactory function when placed in the esophageal bed as described by Thomas et al. [8]

Various organs have been used for esophageal replacement, including the stomach, colon, and jejunum. The choice of conduit depends on patient factors, prior surgeries, and intraoperative findings. The colon is frequently selected because of its adequate length, luminal diameter, and resistance to gastric acid and pepsin, along with its long-term durability when mobilized on a robust vascular arcade. The colon's blood supply derives from the superior and inferior mesenteric arteries, linked by the marginal artery of Drummond, which ensures continuity of perfusion [9]. The middle colic, left colic, and marginal arteries are particularly crucial for maintaining the viability of the conduit.

The technique of right colon conduit has been described by Rice et al. [10]. However, due to possible technical reasons, the terminal part of the colon conduit was congested after harvest. But since the serosa on scoring oozed bright red blood, we proceeded with the right colon conduit with colo-esophageal, colo-jejunal, and ileo-colonic anastomoses.

Early complications unique to colon interposition include anastomotic leak and graft necrosis. Anastomotic leaks occur in 2%-22% of cases, usually at the esophagocolic anastomosis, and result from factors such as ischemia, technical error, tension, infection, or distraction from swallowing and neck movement. Vascular compromise, mainly from venous congestion or thrombosis, may lead to colon graft necrosis in up to 7% of patients [10].

On routine neck exploration on postoperative day 2, we found the colon unhealthy. We removed the right colonic conduit into the abdomen. We then dismantled colo-jejunal anastomoses, resected the necrosed terminal part of the right colon, dismantled the colo-ileal anastomosis, and performed colo-colic anastomosis. We now measured the length of colon required, and we fell short of adequate length if we had to perform a left colic vessels-based conduit. The only option left was to perform an antiperistaltic left colonic conduit based on the middle colic vessels. The technique has been described by Pai et al. [2]. Though it is believed that an isoperistaltic colon conduit is the best option, in this patient, an antiperistaltic left colon conduit was the only available choice, and we proceeded with the same, and the conduit reached the pharynx without any tension. We completed the anastomoses (colo-esophageal, colo-jejunal, and ileo-rectal).

While the retrosternal route may be preferred for a staged colonic conduit, we used the posterior mediastinal route since it offers the shortest route and obviates the necessity for manubrial, clavicle, and rib resection of the thoracic outlet to create space for the colonic conduit [11]. Multivariate analysis identified the conduit position in the posterior mediastinum as an independent predictor of good functional result, as described by Thomas et al. [12].

Although colon conduits are associated with higher morbidity rates than gastric conduits, the long-term outcomes of colon conduits are considered acceptable. The difference in mortality between the groups is likely attributable to underlying medical conditions rather than the surgical technique employed. Furthermore, during the perioperative period, more consideration should be given to the use of a colon conduit, particularly in cervical anastomosis, as described by Kim et al. [13].

## Conclusions

Corrosive-induced multiple esophageal strictures present significant reconstructive challenges requiring meticulous surgical planning and execution. Colonic interposition continues to be a dependable and versatile option for esophageal replacement due to its length, resistance to acid, and stable vascular anatomy. However, early recognition of conduit ischemia remains critical to preventing catastrophic outcomes. When an ascending colonic conduit fails, prompt re-exploration and reconstruction using an antiperistaltic left colonic conduit based on the middle colic artery can provide an effective salvage solution in this challenging clinical situation. Modern surgical experiences indicate that this antiperistaltic configuration does not typically cause significant postoperative functional issues or the severe spasms historically feared in antiperistaltic grafts. This case reinforces the importance of intraoperative vascular assessment, surgical adaptability, and timely intervention in ensuring successful outcomes in complex esophageal reconstructions. We want to note that a Roux-en-Y colojejunal anastomosis may prevent bile reflux into the colon conduit, which we noted in our patient.

## References

[1] Conner A, Raja S ( 2025). Alternative conduits and routes of esophageal reconstruction when a hostile mediastinum is encountered. Foregut: The Journal of the American Foregut Society.

[2] Pai E, Kumar T (2020). Technique of antiperistaltic left colonic conduit. Indian J Surg.

[3] Zargar SA, Kochhar R, Mehta S, Mehta SK (1991). The role of fiberoptic endoscopy in the management of corrosive ingestion and modified endoscopic classification of burns. Gastrointest Endosc.

[4] Gambardella C, Allaria A, Siciliano G (2018). Recurrent esophageal stricture from previous caustic ingestion treated with 40-year self-dilation: case report and review of literature. BMC Gastroenterol.

[5] Gupta V, Wig JD, Kochhar R (2009). Surgical management of gastric cicatrisation resulting from corrosive ingestion. Int J Surg.

[6] Ananthakrishnan N, Nachiappan M, Subba Rao KS (2001). Island pectoralis major myocutaneous flap for pharyngo-oesophageal strictures prior to oesphagocoloplasty. J R Coll Surg Edinb.

[7] Ananthakrishnan N, Parthasarathy G, Maroju NK, Kate V (2007). Sternocleidomastoid muscle myocutaneous flap for corrosive pharyngoesophageal strictures. World J Surg.

[8] Thomas P, Fuentes P, Giudicelli R, Reboud E (1997). Colon interposition for esophageal replacement: current indications and long-term function. Ann Thorac Surg.

[9] Mann MR, Kawzowicz M, Komosa AJ (2021). The marginal artery of Drummond revisited: a systematic review. Transl Res Anat.

[10] Rice TW (1999). Right colon interposition for esophageal replacement. Oper Tech Thorac Cardiovasc Surg.

[11] DeMeester DeMeester, Steven R (2006). Colonic interposition for benign disease. Oper Tech Thorac Cardiovasc Surg.

[12] Thomas P, Giudicelli R, Fuentes P, Reboud E (1996). [Technique and results of colonic esophagoplasties]. Ann Chir.

[13] Kim JH, Yun JK, Kim CW, Kim HR, Kim YH (2024). Long-term outcomes of colon conduits in surgery for primary esophageal cancer: a propensity score-matched comparison to gastric conduits. J Chest Surg.

